# The emergence of waves in random discrete systems

**DOI:** 10.1038/s41598-016-0022-3

**Published:** 2016-12-23

**Authors:** John Pickton, Keith Iain Hopcraft, Eric Jakeman

**Affiliations:** University of Nottingham, School of Mathematical Sciences, Nottingham, NG7 2RD UK

## Abstract

Essential criteria for the emergence of wave-like manifestations occurring in an entirely discrete system are identified using a simple model for the movement of particles through a network. The dynamics are entirely stochastic and memoryless involving a birth-death-migration process. The requirements are that the network should have at least three nodes, that migration should have a directional bias, and that the particle dynamics have a non-local dependence. Well defined bifurcations mark transitions between amorphous, wave-like and collapsed states with an intermittent regime between the latter two.

## Introduction

Manifestations of wave behaviours are so ubiquitous and familiar that the underlying conditions enabling the disturbances to become established and persist may be taken for granted. These considerations are particularly relevant for systems of fundamentally discrete nature^1–7^ where the consequences that obtain from a continuum description cannot be assumed. This prompts enquiring what essential ingredients are required to describe wave disturbances. Before these can be identified, there must first be some ‘medium’ through which an ‘attribute’, be it energy, information, force… is conveyed in a defined direction. Moreover, that attribute must couple dynamically to the medium to account for the action and reaction of one upon the other. The concept of the ‘field’ is especially effective in achieving this aspect. Fields can combine both the medium and attribute within the same theoretical construct, and facilitate action or influence to occur at a distance. Indeed, once a field-theory is formulated, wave behaviours follow with almost inevitable consequence. The second issue is how, once initiated, the system can maintain a persistent state of coherent disturbance in the absence of *coherent* stimulation or any other forcing by an external agency. This then leads to seeking wave-like behaviours as an emergent quality, arising from intrinsic fluctuations that are affected by the structural configuration of the system and the dynamics that occur upon it. The purpose of this article is to identify necessary criteria for achieving this in isolated systems where both medium and attribute have a discrete nature and where the dynamics at the most fundamental level is entirely incoherent, stochastic and memoryless.

One fundamental representation of a discrete medium is a network^8–12^, being an arrangement of sites or nodes that are connected by edges, via which attributes may move from one node to another. The topology of the nodes and edges defines the spatial structure. The nodes are populated by particles that are a discrete representation of the attributes. For a discrete system it is a collection or ensemble of objects rather than the entities themselves (be they tangible particles or more abstract notions like an ensemble of states) that exhibit the characteristics of a wave-like perturbation from equilibrium^13–20^. The qualities exhibited by the disturbance from that equilibrium is described by the dynamics that govern the number of particles at a node, how they move from one node to another, and how these interact with the local and global constraints to which the particles are subjected.

**Figure 1:**
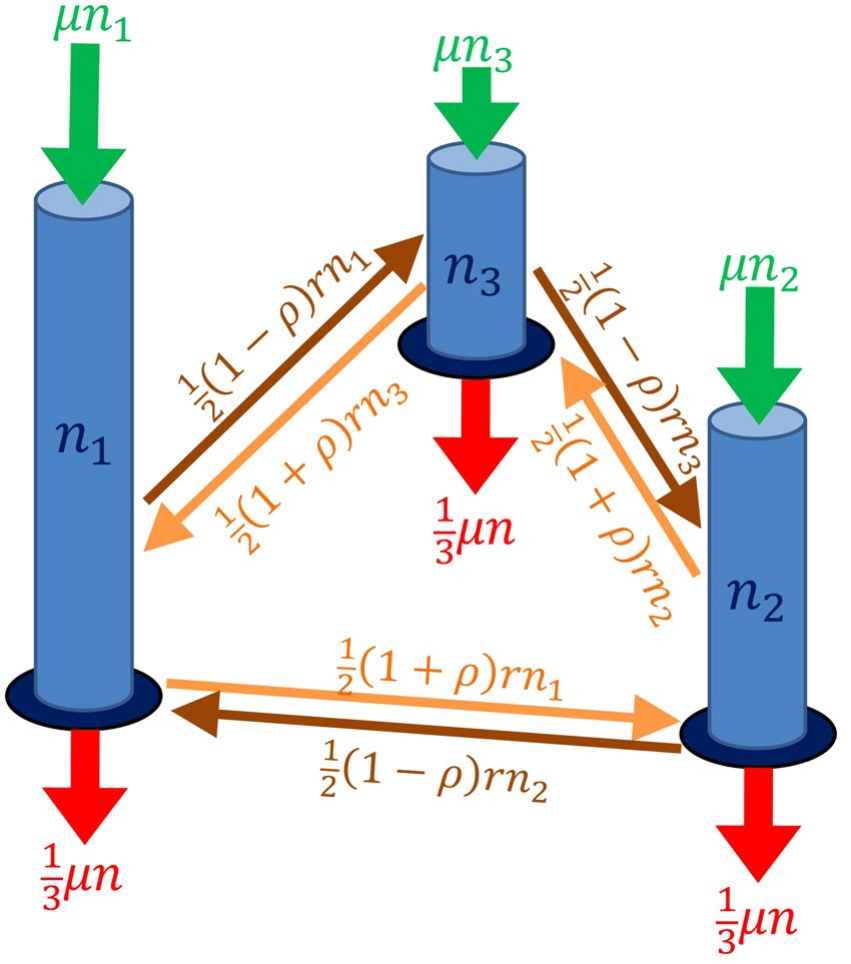
Illustration of the dynamics. Bars (blue) represent populations at each of the three nodes of the network. Small arrows correspond to migration rates of particles between nodes. Large arrows depict the rate of births and deaths of particles at each node.

The system investigated here is not intended to model a specific problem but rather to possess generic features that can be abstracted to particular instances. The dynamics of the system is depicted in Fig. 1 and is described by a Markov stochastic process^21–23^ comprising three elements, each of which can be identified with a key ingredient for emergent wave phenomena. The first element of the dynamics describes the movement of non-interacting particles, spontaneously migrating between the nodes of the network at a constant rate. The response and subsequent evolution of this system to an initial perturbation from equilibrium (be it static or dynamic) is determined by the spectrum of its eigenvalues, one or more of which must have an imaginary part for wave behaviour to ensue. This requires that the chosen network comprise three or more nodes and have directed edges^24^. The simplest network possessing a complex eigenvalue on which this dynamic occurs is a closed loop formed from three nodes. This ‘atom’ therefore provides an essential ingredient upon which to build. And build we must, for the eigenvalues for such dynamics possess a negative real part causing any disturbances to decay. The incoherent and non-interacting nature of this migration process is dissipative and would lead to a dynamic equilibrium with all nodes uniformly populated on average. The second element of the dynamics is a local birth-process^25^ whereby a single particle is added to a node at a rate proportional to the population at that node, characteristic of each particle independently giving birth at a constant rate. The birth-process provides a crucial imbalance in the populations at individual nodes by increasing the real part of the system’s eigenvalues. This thereby modulates the migration of particles and can cause disturbances from equilibrium to grow. However a consequence of the birth-process is that the total population in the entire system will increase exponentially. The third and final element of the dynamics is a global death-process^25^ that is introduced to balance the birth-process by removing particles from the entire system at an equal rate. However unlike the birth-process, which is defined locally at each node, the deaths occur with equal probability from each populated node and are therefore defined globally over the entire network. This mechanism also instils coherence upon the system because each node is affected by the entire ensemble - the death process contains that property that is imbued by a field, albeit through being manifested stochastically. When combined, these simple elements can produce a propagating and persistent coherent disturbance despite the underlying dynamics being entirely incoherent. This model proves to be surprisingly rich by virtue of containing two distinct bifurcations as a single control parameter changes, and these mark the transition between three distinct collective states: amorphous, wave-like and collapsed with an intermittent transitory phase between the latter two.

## Results

### The model and its behaviours

We consider non-interacting particles that are constrained to a network of three nodes forming a closed loop. At any instant the *i* ^*th*^ node is populated by *n*_*i*_ particles, one of which ‘migrates’ via a shared edge to an adjacent node at rate *rn*_*i*_ with probability (1 + *ρ*)/2 in one direction and (1 − *ρ*)/2 in the other, where −1 ≤ *ρ* ≤ 1 provides a bias to the direction of migration; when *ρ* = 0 there is no directional preference. The dynamics at each node is augmented by a birth-death-process that increases and decreases the population at that node by unity with rates *μn*_*i*_ and *μZ* ^−1^(*n*_1_ + *n*_2_ + *n*_3_) respectively, where *Z* is the number of nodes that are populated at that instant - the death process cannot operate at an empty node. The death rate can be interpreted as sampling particles from the system at a total rate *μ*(*n*_1_ + *n*_2_ + *n*_3_) with *equal probability* from each *non-empty* node. The factor of *Z* ^−1^ ensures that the *total* number of particles in the system follows a simple birth-death-process with equal birth- and death-rates^26^. The fine-tuning of the birth and death rates is not critical. Indeed the rates may differ through the introduction of a (Poisson) immigration process provided the death-rate exceeds the birth-rate^26^. However this introduces an extrinsic agency (albeit an incoherent one) to the system, and we are concerned with the emergence of order from intrinsic fluctuations - the detail will be different but the overall outcome similar.

**Figure 2:**
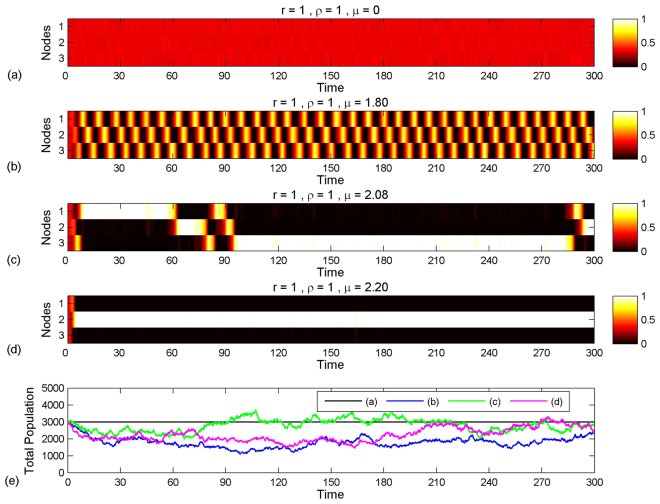
Heat plot of simulated data for four different values of the birth rate *μ* with common migration rate *r* = 1, preference *ρ* = 1. Each simulation initiated with a population of 1000 particles at each node. Colour intensities in the rows correspond to the proportion of particles at each of the three nodes with the scales being given on the right. (**a**) Amorphous-state, *μ* = 0. (**b**) Wave-state, *μ* = 1.8 (*θ* = 0.6). (**c**) Intermittent-state, *μ* = 2.08 (*θ* = 1.16). (**d**) Collapsed-state, *μ* = 2.2 (*θ* = 1.4). The control parameter *θ* = (2*μ* − 3*r*)/*rρ* is discussed in the analysis. (**e**) Total populations in each of the four simulations.

The system at a time *t* is specified by the stochastic configuration vector ${\bf{n}}(t)=({n}_{1}(t),{n}_{2}(t),{n}_{3}(t))$, whose evolution can be envisaged as a random walk on a three-dimensional lattice whose axes are the, non-negative, number of particles at each node. The probabilities *P*_*abc*_(*t*) = Pr{**n**(*t*) = (*a,b,c*)} of being in a particular state are completely determined by the *master equation* ^27,28^,

1 $$\frac{d}{dt}{P}_{ijk}(t)=\sum _{a,b,c}{{\rm{\Gamma }}}_{ijk}^{abc}{P}_{abc}(t),$$

given some initial condition. Here ${{\rm{\Gamma }}}_{ijk}^{abc}$ is the transfer rate from the state $(a,b,c)\to (i,j,k)$ and $-{{\rm{\Gamma }}}_{abc}^{abc}$ is the total transfer rate out of the state (*a,b,c*). Supplementary Note 1 defines these rates explicitly. Insight into the behaviour of the system is gained from Monte-Carlo simulated realizations^29^ of the dynamics for various parameter values. Figure 2 shows simulation results for a network with *ρ* = 1, *r* = 1 and values of *μ* taken in increasing order. Simulations are performed using the Gillespie algorithm^30^, which is exact for Markov processes, see Supplementary Note 2. The qualitative behaviour of the system is similar for all |*ρ*| > 0. For systems with relatively small rates of birth and death, the diffusive nature of the migration process dominates the overall behaviour of the system and the particles settle into an *amorphous-state*, spread uniformly between the three nodes of the network, seen in Fig. 2(a). Increasing *μ* results in an increased correlation between particles and causes larger fluctuations about this equilibrium. However when the birth and death rates are sufficiently large, the correlation between particles begins to manifest at the macroscopic level. The system undergoes a transition and enters a regime of *wave-states* where particles move in coherent fashion around the network, the population at each node periodically emptying and filling with particles, as shown in Fig. 2(b). Wave-like behaviour is shown to emerge, and persist, from an initial state where particles are uniformly spread around the network. Figure 2(e) shows that this collective phenomenon is not evident in the time evolution of the total population in the entire system. Further increase of the birth and death rates causes the system to enter an *intermittent-state* where the wave-behaviour is disrupted by particles temporarily lodging at single nodes, see Fig. 2(c). Increasing the birth and death rates further eventually results in a *collapsed-state* with the bulk of the particles populating a single node, Fig. 2(d). The following sections explain these phenomena and their transitions quantitatively.

### Analysis of the dynamics

To explain the various behaviours of the system we use equation (1) to derive deterministic (macroscopic) equations that provide a first-order approximation of the system in the large population limit, *n*(*t*) ≫ 1 ^27^. This is done by considering the evolution mean population size at each nodes. The mean, denoted by 〈·〉, is an ensemble average of all available states, see the Supplementary Method 1 for details. The stochastic nature of this system plays an important role in the derivation of the equations describing the evolution of the mean populations, due to the death process being dependent on the number of populated nodes. While it is possible to obtain higher order approximations of the system, however the deterministic equations successfully capture the types of behaviours that are observed in simulations.

Consideration of the mean dynamics reduces the dimension of the system because the total population remains constant on average, due to the equal rate of births and deaths. The system evolves as a trajectory in the two-dimensional phase-space, whose geometry forms a triangle given by

2 $$\langle {n}_{1}(t)\rangle +\langle {n}_{2}(t)\rangle +\langle {n}_{3}(t)\rangle =\bar{n},\quad \quad \langle {n}_{i}(t)\rangle > \mathrm{0,}$$

where $\bar{n}={n}_{1}\mathrm{(0)}+{n}_{2}\mathrm{(0)}+{n}_{3}\mathrm{(0)}$ is the *initial* total population in the system. The interior of this phase-space corresponds to states where all nodes of the network are populated, the boundaries to when one of the nodes is depleted and the vertices to when all particles are located at a single node.

The derivation of the equation for the evolution of the mean populations 〈**n**(*t*)〉 is given in the Supplementary Method 1. We can treat the dynamics in the interior and on the boundaries separately. For describing the evolution in the interior it is useful to make the assumption that all of the nodes of the network are populated, i.e. *n*_*i*_ > 0. The equation for the mean populations is then

3 $$\frac{d}{dt}\langle {\bf{n}}(t)\rangle =\langle {\bf{n}}(t)\rangle {{\bf{B}}}_{interior}(r,\rho ,\mu )\equiv \langle {\bf{n}}(t)\rangle ({\bf{M}}(r,\rho )+\mu {{\bf{I}}}_{3}-\,\frac{1}{3}\mu {{\bf{U}}}_{3}),$$

where **B**_*intrerior*_ is a 3 × 3 matrix formed from **I**_3_ the identity matrix that describes births, **U**_3_ with all elements unity which describes the death-process, and

4 $${\bf{M}}(r,\rho )\equiv \frac{1}{2}r \left(\begin{array}{ccc}-\,2 & 1+\rho  & 1-\rho \\ 1-\rho  & -\,2 & 1+\rho \\ 1+\rho  & 1-\rho  & -\,2\end{array}
\right)$$

that accounts for the migrations. By symmetry, equation (3) has an equilibrium point at the centre of the phase-space $\frac{1}{3}(\bar{n},\bar{n},\bar{n})$ which corresponds to particles being uniformly spread around the network, as in the amorphous-state. Indeed this configuration is an eigenvector of **B**_*intrerior*_ with associated eigenvalue *σ*_0_ = 0. The behaviour of the system about this equilibrium is then determined by the two remaining eigenvalues which form a complex conjugate pair

5 $${\sigma }_{\mathrm{1,2}}\equiv \mu -\,\frac{3}{2}r\pm i\frac{\sqrt{3}}{2}r\rho \mathrm{.}$$

Trajectories follow exponential spirals centred at the equilibrium point. The real part of the eigenvalues determines whether the trajectory on the spiral is towards or away from the equilibrium point. The imaginary part of the eigenvalues gives the angular frequency of the trajectory about the equilibrium point, which is zero when there is no directional preference to the migrations, *ρ* = 0.

**Figure 3:**
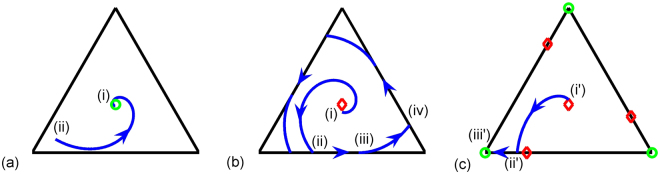
Trajectory of the mean state in the phase-space. Circles (green) represent stable equilibrium points. Diamonds (red) represent unstable equilibrium points. (**a**) Amorphous-state - points (i) and (ii) are starting and finishing points respectively. (**b**) wave-state - (i) is the starting point, (ii) is where the second node empties, (iii) is where the population in the second node begins to repopulate and (iv) is where third node empties. (**c**) collapsed-state - (i′) is the starting point, (ii′) is where the second node empties and (iii′) is where all particles are at the third node.

For birth-death rates satisfying $\mu < \frac{3}{2}r$, the real part the eigenvalues is negative so the equilibrium point is *stable* and trajectories are directed towards the centre of the phase-space, see Fig. 3(a), and realizations of the system evolve to the *amorphous-state* with particles spread uniformly around the network, Fig. 2(a).

When $\mu =\frac{3}{2}r$ the system undergoes a *Hopf bifurcation* ^31,32^, that marks the transition to the *wave-state*. Increasing the birth-death rates so that $\mu > \frac{3}{2}r$, the equilibrium point of equation (3) becomes *unstable* and trajectories in the phase-space spiral *away* from the centre and towards the boundaries. At the boundary equation (3) is no longer valid and an equation to describe the evolution of the system on the boundaries is required. Because each boundary will have an equivalent equation, we suppose that the system reaches the boundary where 〈*n*_2_〉 = 0. By assuming that *n*_2_ = 0 we deduce the equation

6 $$\frac{d}{dt}\langle {\bf{n}}(t)\rangle =\langle {\bf{n}}(t)\rangle {{\bf{B}}}_{boundary}(r,\rho ,\mu )\equiv \langle {\bf{n}}(t)\rangle ({\bf{M}}(r,\rho )+\mu {{\bf{I}}}_{3}-\,\frac{1}{2}\mu {{\bf{U}}}_{2})\mathrm{,}$$

where **U**_2_ is the **U**_3_ matrix with its second column replaced be zeros, and describes the global death-process that occurs at the still-populated nodes - note the pre-factor $\frac{1}{2}$. To describe the evolution of the trajectory in the vicinity of the boundary, equation (6) which is valid when *n*_2 _= 0, must be combined with equation (3), which is valid when *n*_2_ > 0, to account for the random fluctuations of the number of particles at the second node. The evolution of the mean state 〈**n**(*t*)〉 at the boundary is then described by a weighted sum of (3) and (6), the weights being *w* and 1 − *w* respectively where *w* = Pr{*n*_2_ > 0}. A condition for the trajectory to move along the boundary is that 〈*n*_2_〉 remains at 0, so that $\frac{d}{dt}\langle {n}_{2}\rangle =0$. This can be used to determine *w* as:

7 $$w=\frac{3r}{2\mu }(2\rho \frac{\langle {n}_{1}\rangle }{\bar{n}}+1-\rho )\mathrm{.}$$

An alternative derivation of this equation is given in the Supplementary Method 1. The requirement 0 ≤ *w* ≤ 1 implies that equation (7) has a solution when

8 $$\frac{\langle {n}_{1}\rangle }{\bar{n}}\le \frac{1}{2}(1+\frac{\theta }{3})\equiv {\kappa }_{b},$$

where *θ* = (2*μ* − 3*r*)/*rρ* serves as a control parameter involving all three dynamical parameters of the system. At points on the boundary such that $\langle {n}_{1}\rangle > {\kappa }_{b}\bar{n}$, the trajectory evolves according to equation (3) and leaves the boundary - the population *n*_2_ growi*n*g away from zero. However, at points such that $\langle {n}_{1}\rangle < {\kappa }_{b}\bar{n}$ the trajectory is confined to the vicinity of the boundary as the population *n*_2_ is maintained near zero. The evolution of the system along the boundary is then given by

9 $$\frac{d}{dt}\frac{\langle {n}_{1}\rangle }{\bar{n}}=\frac{r\rho \theta }{2}[\frac{\langle {n}_{1}\rangle }{\bar{n}}-\,\frac{1}{2}(1-\frac{1}{\theta })]\mathrm{.}$$

An equation for the population at the third node can be obtained from $\langle {n}_{3}\rangle =\bar{n}-\langle {n}_{1}\rangle$. The direction in which the trajectory moves along the boundary depends on the sign of the right-hand-side of equation (9), which vanishes when 〈*n*_1_〉 has the critical value

10 $$\frac{\langle {n}_{1}\rangle }{\bar{n}}=\frac{1}{2}(1-\frac{1}{\theta })\equiv {\kappa }_{e},$$

a point which corresponds to an *unstable* equilibrium point on the boundary. Consider the trajectory depicted in Fig. 3(b) that is initially at a point (i) and meets the boundary at point (ii) corresponding to where the first and third nodes are occupied, where the right-hand-side of equation (9) is assumed positive. There are two ways in which this can happen. The first occurs unconditionally if 0 < *θ* < 1, the second if *θ* > 1 and $\langle {n}_{1}\rangle > {\kappa }_{e}\bar{n}$. The rate of change of 〈*n*_1_〉 is then positive, so that 〈*n*_1_〉 will increase, and 〈*n*_3_〉 decrease, until the condition (8) is violated at point (iii). The trajectory then leaves the boundary of the phase space, and is once again described by equation (6) until it intersects the next boundary at (iv), corresponding to where only the first and second nodes are populated. Provided that at point (iv) $\langle {n}_{2}\rangle > {\kappa }_{e}\bar{n}$, the trajectory will behave as before, leading to a limit cycle being established that represents a wave of particles travelling around the network. The realization in Fig. 2(b) shows this coherent disturbance emerging out of an initial amorphous-state. Supplementary Video 1 demonstrates the corresponding time evolution dynamically, in both the phase-space and in a histogram showing the number of particles at each node.

Consider now the case depicted in Fig. 3(c) which can also occur if *θ* > 1. The trajectory of the system is initiated from the state (i′), intersecting the boundary at (ii′) where $\langle {n}_{1}\rangle < {\kappa }_{e}\bar{n}$, which corresponds to the right-hand-side of equation (9) being negative. The rate of change of 〈*n*_1_〉 is negative, with result that the trajectory moves in the direction of increasing 〈*n*_3_〉. The trajectory will continue along the boundary until it reaches the vertex of the triangle (iii′) corresponding to only the third node being occupied by particles. The system is then trapped in this collapsed state, as demonstrated by the realization in Fig. 2(d).

Collapsed and wave behaviours can both occur if *θ* > 1. However there is a critical value of *θ* = *θ*_*c*_ above which the wave-state can no longer exist. This corresponds to the value of *θ* for which a limit cycle intersects each boundary at the point where $\langle {n}_{i}\rangle ={\kappa }_{e}\bar{n}$. The Supplementary Method 2 shows that this occurs at the solution of the transcendental equation

11 $$l(\frac{{\theta }_{c}}{\sqrt{3}})+\frac{\pi }{6}=0,$$

where *l*(*x*) ≡ log(*x*)/*x*, the numerical solution of which is *θ*_*c*_ ≈ 1.2037, and consequently a coherent wave-state cannot exist for *θ* above this value. If *θ* > *θ*_*c*_ the system *must* fall into the collapsed-state. If 1 < *θ* < *θ*_*c*_ *both* wave- and collapsed-states can coexist.

Monte-Carlo simulations of the dynamics for values of *θ* in the regime 1 < *θ* < *θ*_*c*_ display intermittent switching between wave- and collapsed-behaviours as shown in Fig. 2(c). This interchange is caused by fluctuations of a realization about the trajectory describing the mean $\langle {\bf{n}}(t)\rangle$. The fluctuations arise because the dynamics are inherently stochastic and discrete, and their magnitude is determined by the number of particles in the system. The intermittent behaviour is not explained by the deterministic equations that provide a leading order approximation to the evolution of the system. The greater the number of particles, the less frequent the interchanges between the macroscopic states will be.

### Characteristics of the wave-state

**Figure 4:**
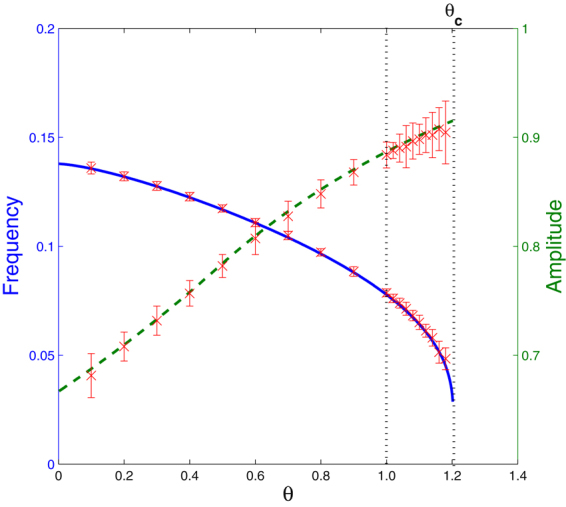
Frequency (solid blue) and amplitude (dashed green) of the limit cycle. Crosses (red) show the estimated means, with the bars giving the estimated standard deviations. The region between the dashed vertical lines indicates the intermittent regime.

The final task is to determine the frequency and amplitude of the oscillations in the regime 0 < *θ* < *θ*_*c*_ for which the wave-states can occur. The wave-states are those whose trajectories in the phase-space spend part of their time within the interior following an exponential spiral, and moving along the boundary following equation (9), or its equivalent depending upon which boundary the motion is occurring. Calculating these residency times enables a frequency *f* and an amplitude $A\equiv \,{\rm{\max }}\,{n}_{1}/\langle n\rangle$ to be determined. Supplementary Method 2 shows that *A* = *A*(*θ*) and $f=r\rho \hat{f}(\theta )$ where *A* and $\hat{f}$ are solutions of transcendental equations requiring numerical evaluation. These scaling functions show that the quantitative value of the amplitude and frequency depend on *θ* alone and that *rρ*, the characteristic rate at which particles migrate around the network in a defined direction, provides a natural scaling for the time. In Fig. 4 the full (blue) line shows the frequency plotted as a function of *θ*, which has a good agreement with the (red) crosses that show the frequency determined from 10 Monte Carlo simulated realisations of the system with the network populated with an initial population of 30,000 particles; the bars on these data are standard deviations. The frequency decreases with increasing *θ*, falling rapidly to zero in the vicinity of *θ* = *θ*_*c*_. Indeed it can be shown that as *θ* → *θ*_*c*_, $\hat{f}(\theta )\sim -\,{(\mathrm{log}|\theta -{\theta }_{c}|)}^{-1}$, indicative of the system condensing into the collapsed state. The amplitude is shown by the dashed (green) line with the (red) crosses and bars showing the values and their standard deviations obtained from the Monte-Carlo simulation. The amplitude increases with *θ* and at *θ* = *θ*_*c*_ changes discontinuously into the collapsed-state. The fluctuations in both *A* and *f* can be seen to grow as *θ* → *θ*_*c*_.

### Extension to an *N*-node loop

The simple model discussed here can be easily extended to more general networks, in particular to larger loop networks with *N* nodes. In this case the two dimensional phase-space given by equation (2) would be replaced by the *N* − 1 dimensional simplex ${\sum }_{i=1}^{N}\langle {n}_{i}\rangle =1$, 〈*n*_*i*_〉 > 0. The evolution in the interior of this simplex, i.e. when all nodes are populated, would be given by an equivalent of equation (3) with the matrices instead being of size *N* × *N*. Again there would be an equilibrium at the centre of the phase-space corresponding to the amorphous-state with all particles being spread uniformly around the network. The stability of this equilibrium would be determined by the non-zero eigenvalues, which come in complex conjugate pairs, given by

12 $${\sigma }_{k}\equiv \mu -r+r\,\cos \,\frac{2\pi k}{N}+ir\rho \,\sin \,\frac{2\pi k}{N},$$

where *k* = 1, 2, …, *N*−1. The equilibrium is stable when all of the eigenvalues have a negative real part and the system settles into an amorphous-state. Simulation results for a system with *N* = 10, *r* = 1 and *ρ* = 1 are shown in the Supplementary Videos 2 and 3. A transition out of the amorphous-state occurs as two of the eigenvalues, with *k* = 1 and *N* − 1, cross the imaginary axis, i.e. if $\mu \ge r\,(1-\,\cos \,\frac{2\pi }{N})$. Thus the transition is independent of the directional preference *ρ*. As the size of the network becomes large, this bifurcation point approaches zero so the transition occurs for progressively smaller birth and death rates. For a network with *N* = 10, *r* = 1 and *ρ* = 1 the transition occurs when $\mu \approx 0.19$ and the Supplementary Video 4 shows such a system just above this bifurcation point. Whether or not the system enters into the regime of wave-states is dependent on the imaginary part of the two eigenvalues. No oscillation occurs when *ρ* = 0 because the eigenvalues are real and the system transitions into a collapsed-state with all of the particles located at a single node. However when *ρ* ≠ 1 wave-like behaviour will emerge in the form of particles moving coherently around the network, demonstrated in the Supplementary Videos 5 and 6 for a network with *N* = 10, *r* = 1 and *ρ* = 1. It is notable that the width of the disturbance reduces with an increasing birth-death rate *μ*.

Explaining the transition of the system out of the wave-states into a collapsed-state would require deriving the equations that govern system at the boundaries of the phase-space. However, by running simulations for various network sizes *N* it is observed that the size of the network does not have a significant effect on the second bifurcation. Supplementary Videos 7, 8 and 9 demonstrate the transition into the collapsed-state for a system with *N* = 10, *r* = 1 and *ρ* = 1, which can be compared with Fig. 2(c) and (d) where *N* = 3, *r* = 1 and *ρ* = 1. This similarity between systems with different sized networks is essentially because the death process, which is the only non-local component to the dynamics, depends on the number of populated nodes. If all of the particles located at a single node, the dynamics are independent of the number of non-populated nodes and the system size would be irrelevant.

## Discussion

The purpose of this article is to use a simple model to identify the essential ingredients required for a discrete attribute to emerge from intrinsic fluctuations as a periodic and self-sustaining disturbance that moves coherently through a discrete medium. The medium is represented by a network of nodes connected by edges, and the attribute is represented by particles that are subjected to a stochastic birth-death-migration process. The migration process describes the coupling between the particles and the network structure and causes particles to diffuse over the network into an amorphous-state. In contrast the births and deaths provide a coupling amongst the particles and cause them to collect into a single node in a collapsed-state.

The combination of migration process and the birth-death process can result in the emergence of wave-phenomena provided the system satisfies two requirements of the spatial structure; the network must comprise at least three nodes and the migrations must have a directional preference. This is because the system must have complex eigenvalues, the imaginary part of which are completely determined by the migrations and is independent of the birth-death process, see (5). No directional preference to migrations corresponds to a symmetric matrix in place of (4) which necessarily has real eigenvalues. Similarly a two node network only has one non-zero eigenvalue which must be real.

The strength of the present model is its simplicity and the insights it provides. Firstly we have chosen to define the dynamics as Markov stochastic processes so that the system does not have any memory of its past. The time intervals between particle migrations, births or deaths are then independent and exponentially distributed. Defining the dynamics differently would imply that the system stores information about its past state and this will impose correlations upon the particle behaviours. A lack of memory does not allow dead/delay times which introduce intrinsic time-scales into the system^33^. The second notable feature of the model is that both the migration and the birth process are characteristic of non-interacting particles - each particle migrates or gives birth at a fixed rate. This means that the resultant effect of these processes at a single node of the network scales linearly with the population size at that node. If instead the rate of migrations from each node were independent of the population size, i.e. constant, the process would no longer be diffusive and would lack the mechanism by which wave-like behaviour is established on the network. Similarly the birth process allows larger populations to grow faster than smaller ones, a key ingredient in preventing the decay of periodic behaviour. The third feature of the model is the mechanism by which action or influence occurs at a distance. In systems with continuous state-spaces this property is often described by a field. In the present model this is facilitated by the death process. The emergence of wave-like behaviour requires non-local information to be available at each node.

The analysis presented in this paper shows that the observed phenomena are well explained by assuming the number of particles in the system is essentially infinite, describing the dynamics macroscopically. The emergence of wave-like behaviour is then characterised by the appearance of a limit cycle in the solution phase-space. However, it has been shown that oscillations my also exist in systems with relatively moderate number of particles even when the macroscopic equations do not have a periodic solution, a phenomena sometimes referred to as ‘amplification’^34,35^. The wave-state is stochastic, emerging from and being sustained by intrinsic fluctuations occurring on the network. Although similar in manifestation, the cause of the wave-state is distinct from that described by stochastic resonance^36,37^. Here a system is coupled to an extrinsic, weak coherent drive whose characteristic frequencies coincide with some eigen-frequencies of the network, i.e. the spectrum of the noise.

Nonlinearity in the dynamics is solely due to the constraints imposed on the death process. An alternative way to introduce nonlinearity is to include quadratic effects, where particles migrate from the *i* ^*th*^ node to the *j* ^*th*^ node at a rate proportional to *n*_*i*_ *n*_*j*_, essentially representing forces between particles. It has been demonstrated that such systems can display wave-like behaviour^38^, providing the interactions are not symmetric. More recently these models have been used to study condensation phenomena^39^ similar to the collapsed-state demonstrated in Fig. 2(d). Thus the present model captures both condensation and wave phenomena without explicitly defining a force between particles.

## Electronic supplementary material

Supplementary Video 1

Supplementary Video 2

Supplementary Video 3

Supplementary Video 4

Supplementary Video 5

Supplementary Video 6

Supplementary Video 7

Supplementary Video 8

Supplementary Video 9

Supplementary Information
