# Supplementary material for: The emergence of waves in random discrete systems
Source: Sci Rep. 2016 Dec 23;6:21. doi: 10.1038/s41598-016-0022-3 (PMC5431357; doi:10.1038/s41598-016-0022-3)
Supplement: Supplementary file 10 — Supplementary Information [file 41598_2016_22_MOESM10_ESM.pdf]

The emergence of waves in random discrete systems (supplementary information)

J. Pickton*, K.I.Hopcraft, E. Jakeman

School of Mathematical Sciences, University of Nottingham, Nottingham, NG7 2RD, UK

pmxjp5@nottingham.ac.uk

SUPPLEMENTARY FIGURE 1

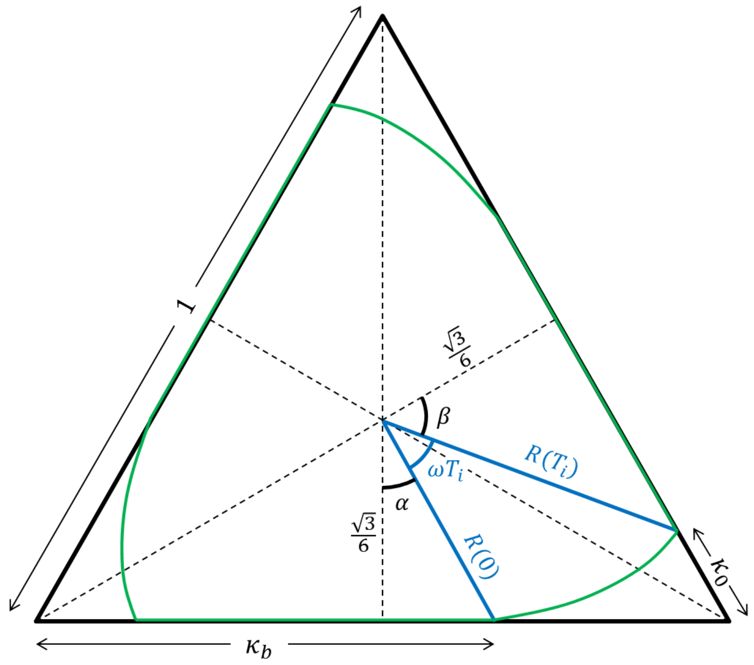

Figure 1: Geometrical picture of the limit cycle. The phase plane has been scaled so that the boundaries have unit length. The cycle meets the boundaries at a point κ_0 and leaves at a point κ_b.

SUPPLEMENTARY FIGURE 2

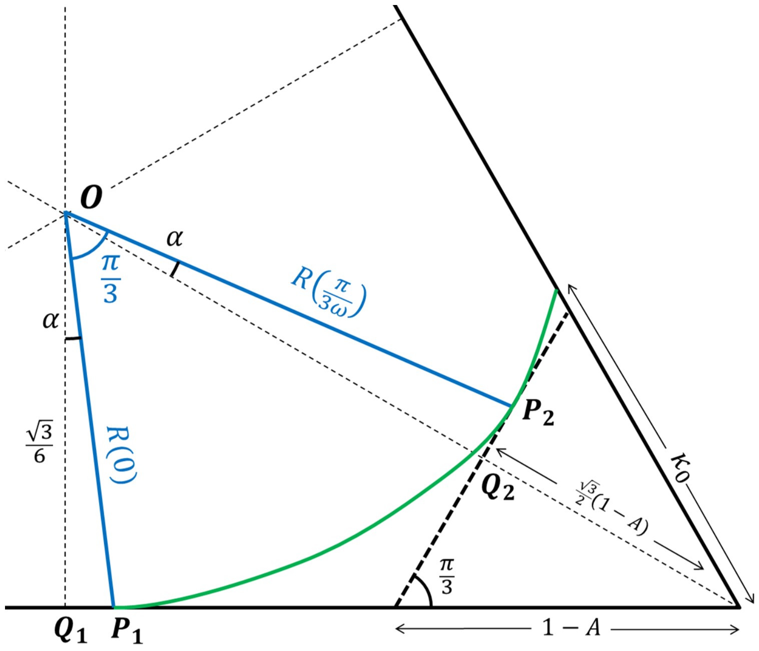

Figure 2: Diagram of one third of the phase-space, used in calculating the amplitude A.

SUPPLEMENTARY FIGURE 3

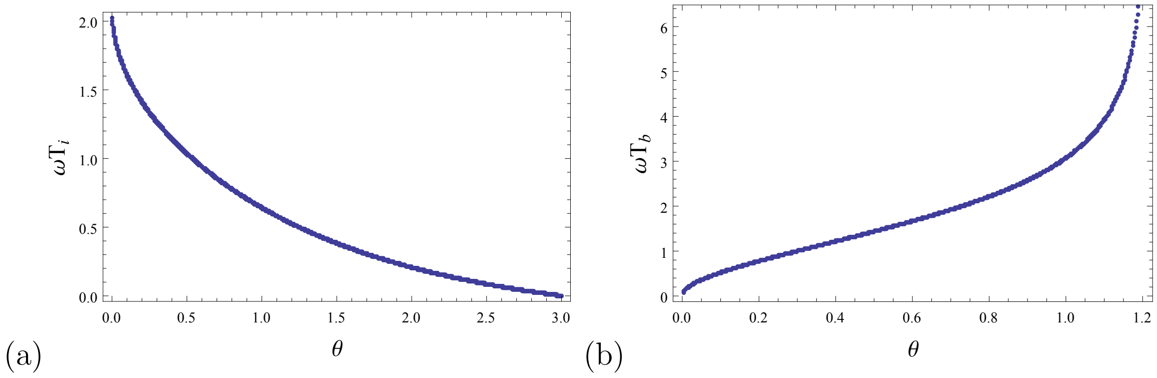

Figure 3: Numerical solutions for (a) ωT_i and (b) ωT_b, plotted against θ. Note how $T_b \to \infty$ as $\theta \to \theta_c$.

SUPPLEMENTARY FIGURE 4

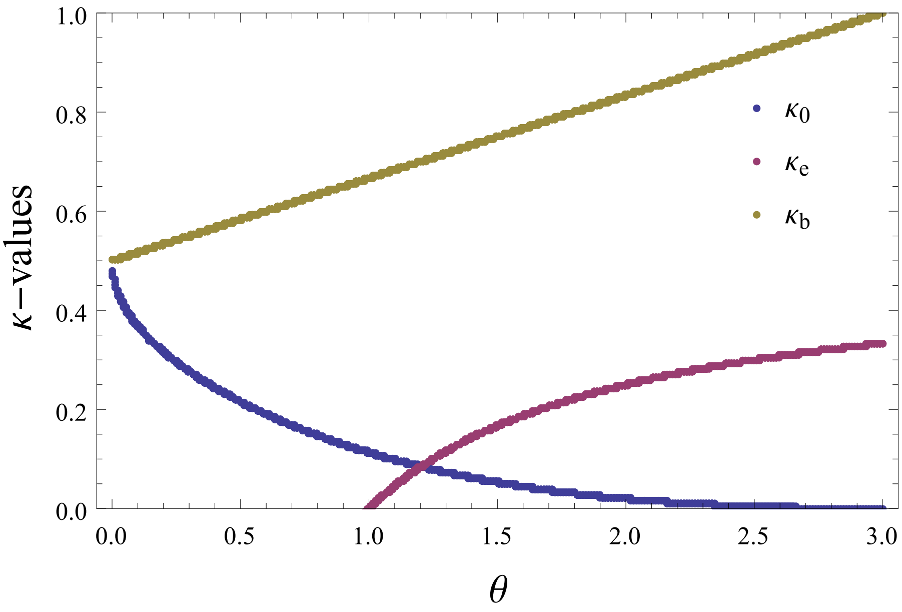

Figure 4: The three values of κ_b, κ_e and κ_0 plotted against θ. Note how $\kappa_e = \kappa_0$ when $\theta = \theta_c$.

SUPPLEMENTARY VIDEO 1

Simulation results of the time evolution of a system with $N = 3$, $r = 1$, $\rho = 1$ and $\mu = 1.8$. The system is initiated with 1000 particles at each node. (Top Left) The height of the bars corresponds to the number of particles at each node. (Top Right) Trajectory of the system in the phase-space. (Bottom) Heat plot of the time evolution.

SUPPLEMENTARY VIDEO 2

Simulation results of the time evolution of a system with $N = 10$, $r = 1$, $\rho = 1$ and $\mu = 0$. The system is initiated with 100 particles at each node. The height of the bars corresponds to the number of particles at each node.

SUPPLEMENTARY VIDEO 3

Simulation results of the time evolution of a system with $N = 10$, $r = 1$, $\rho = 1$ and $\mu = 0.18$. The system is initiated with 100 particles at each node. The height of the bars corresponds to the number of particles at each node.

SUPPLEMENTARY VIDEO 4

Simulation results of the time evolution of a system with $N = 10$, $r = 1$, $\rho = 1$ and $\mu = 0.2$. The system is initiated with 100 particles at each node. The height of the bars corresponds to the number of particles at each node.

SUPPLEMENTARY VIDEO 5

Simulation results of the time evolution of a system with $N = 10$, $r = 1$, $\rho = 1$ and $\mu = 0.5$. The system is initiated with 100 particles at each node. The height of the bars corresponds to the number of particles at each node.

SUPPLEMENTARY VIDEO 6

Simulation results of the time evolution of a system with $N = 10$, $r = 1$, $\rho = 1$ and $\mu = 1$. The system is initiated with 100 particles at each node. The height of the bars corresponds to the number of particles at each node.

SUPPLEMENTARY VIDEO 7

Simulation results of the time evolution of a system with $N = 10$, $r = 1$, $\rho = 1$ and $\mu = 2$. The system is initiated with 100 particles at each node. The height of the bars corresponds to the number of particles at each node.

SUPPLEMENTARY VIDEO 8

Simulation results of the time evolution of a system with $N = 10$, $r = 1$, $\rho = 1$ and $\mu = 2.1$. The system is initiated with 100 particles at each node. The height of the bars corresponds to the number of particles at each node.

SUPPLEMENTARY VIDEO 9

Simulation results of the time evolution of a system with $N = 10$, $r = 1$, $\rho = 1$ and $\mu = 2.2$. The system is initiated with 100 particles at each node. The height of the bars corresponds to the number of particles at each node.

Supplementary Note 1 : Master Equation

The purpose of this section is to explicitly define the master equation for the dynamics considered in the article. Define $P_{abc}(t)$ to be the probability of being in the state $\mathbf{n}(t) = (a, b, c)$ at time t and the rate at which the system moves from (a, b, c) to (i, j, k) as Γ^{abc}_{ijk}. The diagonal term $-\Gamma^{abc}_{abc}$ gives the rate at which the system leaves the configuration (a, b, c). The master equation governing the evolution of the probabilities is

$$\frac{d}{dt}P_{ijk}(t) = \sum_{abc} \Gamma^{abc}_{ijk} P_{abc}(t), \tag{1}$$

where the summation is over all possible configurations. Let $\Gamma^{abc}_{ijk} \equiv \mathcal{M}^{abc}_{ijk} + \mathcal{B}^{abc}_{ijk} + \mathcal{D}^{abc}_{ijk}$, where the $\mathcal{M}$, $\mathcal{B}$ and $\mathcal{D}$ are contributions from the migrations, births and deaths respectively. These terms can be defined explicitly; the contribution from the migrations being

$$\begin{aligned}
&\mathcal{M}^{abc}_{(a-1)(b+1)c} = \frac{1}{2}(1+\rho)ra, \qquad \mathcal{M}^{abc}_{(a-1)b(c+1)} = \frac{1}{2}(1-\rho)ra,\\
&\mathcal{M}^{abc}_{a(b-1)(c+1)} = \frac{1}{2}(1+\rho)rb, \qquad \mathcal{M}^{abc}_{(a+1)(b-1)c} = \frac{1}{2}(1-\rho)rb,\\
&\mathcal{M}^{abc}_{(a+1)b(c-1)} = \frac{1}{2}(1+\rho)rc, \qquad \mathcal{M}^{abc}_{a(b+1)(c-1)} = \frac{1}{2}(1-\rho)rc,\\
&\mathcal{M}^{abc}_{abc} = -r(a+b+c).
\end{aligned} \tag{2}$$

The contribution from the births is

$$\mathcal{B}^{abc}_{(a+1)bc} = \mu a, \qquad \mathcal{B}^{abc}_{a(b+1)c} = \mu b, \qquad \mathcal{B}^{abc}_{ab(c+1)} = \mu c, \qquad \mathcal{B}^{abc}_{abc} = -\mu(a+b+c). \tag{3}$$

The contribution from the deaths is

$$\begin{aligned}
&\mathcal{D}^{abc}_{(a-1)bc} = \mathcal{D}^{abc}_{a(b-1)c} = \mathcal{D}^{abc}_{ab(c-1)} = \frac{1}{3}\mu(a+b+c), \qquad \text{when } a, b, c > 0,\\
&\mathcal{D}^{a0c}_{(a-1)0c} = \mathcal{D}^{a0c}_{a0(c-1)} = \frac{1}{2}\mu(a+c), \qquad \text{when } a, c > 0,\\
&\mathcal{D}^{ab0}_{(a-1)b0} = \mathcal{D}^{ab0}_{a(b-1)0} = \frac{1}{2}\mu(a+b), \qquad \text{when } a, b > 0,\\
&\mathcal{D}^{0bc}_{0(b-1)c} = \mathcal{D}^{0bc}_{0b(c-1)} = \frac{1}{2}\mu(b+c), \qquad \text{when } b, c > 0,\\
&\mathcal{D}^{a00}_{(a-1)00} = \mu a, \qquad \text{when } a > 0,\\
&\mathcal{D}^{0b0}_{0(b-1)0} = \mu b, \qquad \text{when } b > 0,\\
&\mathcal{D}^{00c}_{00(c-1)} = \mu c, \qquad \text{when } c > 0,\\
&\mathcal{D}^{abc}_{abc} = -\mu(a+b+c).
\end{aligned} \tag{4}$$

Supplementary Note 2 : MATLAB code

The following MATLAB code is used to generate simulations using the Gillespie Algorithm.

```
function[ output ] = BDM_Loop( N, r, mu, rho, Pops1, T)

%% Description

% BDM_Loop_1(N,r,mu,rho,Pops1,T) returns a data structure containing
% simulations of a birth-death-migration process on a loop network.
%
% N     := Network size
% r     := Migration rate
% mu    := Birth rate
% rho   := Preference of migratations
% Pops1 := Initial populations
% T     := Number of events to be simulated
%
% Defult parameters:
% N = 3
% r = 1
% mu = 0
% rho = 1
% Pops1 = (100,100,...,100)
% T = 10^5

%% Defult parameters

if nargin == 0
    N = 3 ;
    r = 1 ;
    mu = 0 ;
    rho = 1 ;
    Pops1 = ones(1,N)*100;
    T = 10^5 ;

elseif nargin == 1
    r = 1 ;
    mu = 0 ;
    rho = 1 ;
    Pops1 = ones(1,N)*100;
    T = 10^5 ;

elseif nargin == 2
    mu = 0 ;
    rho = 1 ;
    Pops1 = ones(1,N)*100;
    T = 10^5 ;

elseif nargin == 3
    rho = 1 ;
    Pops1 = ones(1,N)*100;
    T = 10^5 ;
```

```
elseif nargin == 4
    Pops1 = ones(1,N)*100;
    T = 10^5 ;

elseif nargin == 5
    T = 10^5 ;

end


%% Creating variables for data storage

Pops = zeros(T+1,N) ; % Populations matrix
Pops(1,:) = Pops1 ;

n = zeros(T+1,1) ; % Total populations vector
n(1) = sum(Pops(1,:));

times = zeros(T+1,1) ; % Event times vector


%% Creating the migration transition matrix

pf = (1+rho)/2 ; % Probability of forward migration
pb = (1-rho)/2 ; % Probability of backward migration
MP = zeros(N) ; % Transition matrix
for i=1:N
    MP(i,:) = circshift([0,pf,zeros(1,N-3),pb],[0,i-1]) ;
end


%% Types of events and their associated probabilities

% Event types
types = {'birth' , 'death' , 'migration'};
% Probabilities
w = zeros(1,3);
w(1) = mu / (2*mu+r) ;
w(2) = mu / (2*mu+r) ;
w(3) = r / (2*mu+r) ;
% These are used a weights when choosing which type of event takes place

%% Simulating the populations and times

for i = 1:T
    Pops(i+1,:) = Pops(i,:); % Initialise the population
    type = types(randsample(1:3,1,true,w)); % choosing event type

    if strcmp(type,'birth')
        node = randsample(1:N,1,true,Pops(i,:)); % choosing node
        Pops(i+1,node) = Pops(i,node) + 1; % adding particle

    elseif strcmp(type,'death')
        weight = Pops(i,:) > 0 ; % all nodes with one or more particles
```

```
        node = randsample(1:N,1,true,weight); % choosing node
        Pops(i+1,node) = Pops(i,node) - 1; % removing particle

    elseif strcmp(type,'migration')
        node1 = randsample(1:N,1,true,Pops(i,:)); % choosing exit-node
        Pops(i+1,node1) = Pops(i,node1) - 1; % removing particle
        node2 = randsample(1:N,1,true,MP(node1,:)); % choosing in-node
        Pops(i+1,node2) = Pops(i,node2) + 1; % adding particle
    end

    n(i+1) = sum(Pops(i+1,:)); % Total population
    % The time between events is exponentially distributed with a rate
    % equal to (2*mu+r)*n(i)
    times(i+1) = times(i) + exprnd(1,1,1)/((2*mu+r)*n(i)) ; % event times
end

%% Creating a data structure containing the results

output = struct('NetworkSize',N,'MigrationRate',r,'BirthRate',mu,...
    'Preference',rho,'Populations',Pops,'Times',times);
```

Supplementary Method 1 : Expected populations

This section uses the master equation to derive the equations for the mean configurations $\langle \mathbf{n}(t) \rangle$. The mean population size at each node are defined as

$$\langle n_1(t) \rangle = \sum_{ijk} i P_{ijk}(t), \quad \langle n_2(t) \rangle = \sum_{ijk} j P_{ijk}(t), \quad \langle n_3(t) \rangle = \sum_{ijk} k P_{ijk}(t), \tag{5}$$

where the averaging is over all possible configurations at time t. The master equation (1) can be used to obtain the equations that govern the evolution of the ensemble averages,

$$\frac{d}{dt} \langle n_1 \rangle = \sum_{ijk} i \frac{d}{dt} P_{ijk} = \sum_{abc} P_{abc} \sum_{ijk} i \Gamma^{abc}_{ijk} \tag{6}$$

and the other equations follow in a similar fashion.

Contribution from the migration process

Consider first the contribution from the migrations,

$$\begin{aligned}
a\mathcal{M}^{abc}_{abc} &= -r(a^2 + ab + ac), \\
(a-1)\mathcal{M}^{abc}_{(a-1)(b+1)c} &= \frac{1}{2}(1+\rho) r (a^2 - a), \\
(a-1)\mathcal{M}^{abc}_{(a-1)b(c+1)} &= \frac{1}{2}(1-\rho) r (a^2 - a), \\
a\mathcal{M}^{abc}_{a(b-1)(c+1)} &= \frac{1}{2}(1+\rho) r (ab), \\
(a+1)\mathcal{M}^{abc}_{(a+1)(b-1)c} &= \frac{1}{2}(1-\rho) r (ab + b), \\
(a+1)\mathcal{M}^{abc}_{(a+1)b(c-1)} &= \frac{1}{2}(1+\rho) r (ac + c), \\
a\mathcal{M}^{abc}_{a(b+1)(c-1)} &= \frac{1}{2}(1-\rho) r (ac), \\
\frac{1}{r} \sum_{ijk} i\mathcal{M}^{abc}_{ijk} &= -a + \frac{1}{2}(1-\rho) b + \frac{1}{2}(1+\rho) c.
\end{aligned} \tag{7}$$

Thus in the absence of the births and deaths, (6) combined with (5) gives,

$$\frac{1}{r} \frac{d}{dt} \langle n_1 \rangle = -\langle n_1 \rangle + \frac{1}{2}(1-\rho) \langle n_2 \rangle + \frac{1}{2}(1+\rho) \langle n_3 \rangle. \tag{8}$$

Similar equations are obtained for the other two populations and all three can be combined into the one matrix equation,

$$\frac{d}{dt}\langle \mathbf{n}(t) \rangle = \langle \mathbf{n}(t) \rangle \mathbf{M}(r, \rho). \tag{9}$$

where $\mathbf{M}$ is defined in the main text.

Contribution from the birth process

Following a similar method to before obtains the effect of the births on the mean populations,

$$\begin{aligned} a\mathcal{B}^{abc}_{abc} &= -\mu(a^2 + ab + ac), \\ (a+1)\mathcal{B}^{abc}_{(a+1)bc} &= \mu(a^2 + a), \\ a\mathcal{B}^{abc}_{a(b+1)c} &= \mu ab, \\ a\mathcal{B}^{abc}_{ab(c+1)} &= \mu ac, \\ \sum_{ijk} i\mathcal{B}^{abc}_{ijk} &= \mu a. \end{aligned} \tag{10}$$

Hence the contribution from the births is simply

$$\frac{d}{dt}\langle n_1 \rangle = \mu \langle n_1 \rangle. \tag{11}$$

From this equation it is clear that the birth process would result in an exponential increase in the number of particles at each node. Similar equations for $\langle n_2 \rangle$ and $\langle n_3 \rangle$ can be combined into the matrix equation

$$\frac{d}{dt}\langle \mathbf{n}(t) \rangle = \langle \mathbf{n}(t) \rangle \mu \mathbf{I}_3. \tag{12}$$

where $\mathbf{I}_3$ is the 3×3 identity matrix.

Contribution from the death process

The death process only involves populated nodes. By proceeding in the same manner as before, the following expression for $\langle n_1(t) \rangle$ describes the contribution from the death process,

$$-\frac{1}{\mu}\frac{d}{dt}\langle n_1 \rangle = \frac{1}{3}\sum_{abc>0} nP_{abc} + \frac{1}{2}\left(\sum_{ab>0} nP_{ab0} + \sum_{ac>0} nP_{a0c}\right) + \sum_{a>0} nP_{a00}, \tag{13}$$

where $n = a + b + c$. This equation involves the probabilities P_{ijk} in a way that cannot be rewritten in terms of the $\langle n_i \rangle$. By making the assumption that all of the nodes are populated the the effect of the death process is

$$\frac{d}{dt}\langle \mathbf{n}(t) \rangle = -\langle \mathbf{n}(t) \rangle \frac{1}{3}\mu \mathbf{U}_3. \tag{14}$$

where the matrix $\mathbf{U}_3$ has all elements equal to one. Combining this with the equations for the migrations and the births results in a the equations used in the interior of the phase space described in the text. When the population at the second node n_2 is equal to zero and the other two populations are non-zero. (13) instead gives

$$\frac{d}{dt}\langle \mathbf{n}(t) \rangle = -\langle \mathbf{n}(t) \rangle \frac{1}{2}\mu \mathbf{U}_2. \tag{15}$$

with $\mathbf{U}_2$ being the $\mathbf{U}_3$ matrix with the second column replaced by zeros. Combining this with the equations for the migrations and the births gives the second equation that is described in the main text, used for explaining the evolution of the system near the boundaries of the phase-space.

The equation describing the motion at the boundary where $\langle n_2 \rangle = 0$ is a weighted sum of (14) and (15) obtained by assuming $n_2 > 0$ and the equation obtained from $n_2 = 0$. This is because the randomness of the system causes it to fluctuate between $n_2 > 0$ and $n_2 = 0$. The appropriate weighting is $w = \Pr\{n_2 > 0\}$ so that at the boundary the contribution from the death process is given by

$$\frac{d}{dt}\langle \mathbf{n}(t) \rangle = -\langle \mathbf{n}(t) \rangle \mu \left[\frac{1}{3} w \mathbf{U}_3 + \frac{1}{2}(1-w)\mathbf{U}_2 \right]. \tag{16}$$

In particular for the mean population in node 1,

$$\frac{d}{dt}\langle n_1 \rangle = \frac{1}{2}\mu \bar{n} \left(\frac{1}{3} w - 1 \right). \tag{17}$$

An alternative derivation for w is provided to that given in the text, which uses the fact that the state characterising $n_2(t)$ undergoes a random walk near the boundary. Increases in $n_2(t)$ due to migrations and births occur at a rate $u \equiv \mu n_2 + r\frac{1}{2}(1+\rho)n_1 + r\frac{1}{2}(1-\rho)n_3$, which can be approximated by noting that $n_1, n_3 >> n_2$ and $n_i \approx \langle n_i \rangle$ in which case $u \approx r\rho\langle n_1 \rangle + r\frac{1}{2}(1-\rho)\bar{n}$. Similarly decreases in $n_2(t)$ due to migrations and deaths occur at a rate $d \equiv \frac{1}{3}\mu n + r n_2$, which may be approximated by $d \approx \frac{1}{3}\mu\bar{n}$. Therefore the population size n_2 at the second node approximately follows an asymmetric random walk, bounded below at zero. The stationary distribution of this process determines the individual probabilities $\Pr\{n_2 = j\}$. When $u > d$ the increases in n_2 dominate decreases, the system moves away from the boundary, the value of w is approximately one and the equations for the interior for the phase-space are sufficient to describe the evolution

of the system. However, when $u < d$ decreases in n_2 dominate increases and there is a stationary distribution of the process. This distribution is geometric with parameter u/d, the individual probabilities given by

$$\Pr\{n_2 = j\} = \left(1 - \frac{u}{d}\right)\left(\frac{u}{d}\right)^j. \tag{18}$$

Hence we have found an estimation to the probability equivalent to that found in the main text,

$$w = \Pr\{n_2 > 0\} = 1 - \Pr\{n_2 = 0\} = \frac{u}{d}. \tag{19}$$

Substituting this into (17) gives the effect of the death process on the mean evolution at the boundary corresponding to $\langle n_2 \rangle = 0$,

$$2\frac{d}{dt}\frac{\langle n_1 \rangle}{\bar{n}} = -\mu + r\left[\rho\frac{\langle n_1 \rangle}{\bar{n}} + \frac{1}{2}(1 - \rho)\right]. \tag{20}$$

The effect from the migrations and the births are respectively,

$$2\frac{d}{dt}\frac{\langle n_1 \rangle}{\bar{n}} = -r(3 + \rho)\frac{\langle n_1 \rangle}{\bar{n}} - r(1 + \rho), \qquad \frac{d}{dt}\frac{\langle n_1 \rangle}{\bar{n}} = \mu\frac{\langle n_1 \rangle}{\bar{n}}. \tag{21}$$

Combining these equations together gives

$$2\frac{d}{dt}\frac{\langle n_1 \rangle}{\bar{n}} = (2\mu - 3r)\left[\frac{\langle n_1 \rangle}{\bar{n}} - \frac{1}{2}\right] + \frac{1}{2}r\rho. \tag{22}$$

Supplementary Method 2 : Calculation of the frequency, amplitude and the critical value θ_c

This section uses the two equations governing the evolution of the mean dynamics in the interior and on the boundaries of the phase space to calculate the frequency and amplitude of the system in its wave-state. These also allow evaluation of the critical value θ_c for the cut-off of the wave state. To determine the frequency of the cycle requires calculating the time T_i spent by the system moving between neighbouring boundaries and the time T_b spent by the system moving along a boundary, achieved by analysing the geometry of the limit cycle in the phase-space. It is useful to scale the lengths so that each edge of the triangular phase-space has unit length, i.e. dividing by a factor of $\bar{n}$. The geometrical picture of the limit cycle in the phase-space is given in Supplementary Fig. 1.

The parameters θ, κ_b and κ_e are defined in the main text and we define δ and ω to be the real and imaginary part of the eigenvalue σ_1 respectively;

$$\delta \equiv \mu - \frac{3}{2}r, \qquad \omega \equiv \frac{\sqrt{3}}{2}r\rho, \qquad \hat{\theta} \equiv \frac{\theta}{\sqrt{3}} = \frac{\delta}{\omega},$$
$$\kappa_b \equiv \frac{1}{2}\left(1 + \frac{\theta}{3}\right) \qquad \text{and} \qquad \kappa_e \equiv \frac{1}{2}\left(1 - \frac{1}{\theta}\right). \tag{23}$$

Calculating the frequency

Evaluation of T_b, the time spent on the boundary, requires the solution of (22), which can be rewritten as

$$\frac{1}{\delta}\frac{d}{dt}\frac{\langle n_1 \rangle}{\bar{n}} = \frac{\langle n_1 \rangle}{\bar{n}} - \kappa_e, \tag{24}$$

with the initial condition $\langle n_1(0) \rangle = \kappa_0 \bar{n}$ corresponding to the point at which the trajectory reaches the boundary. The trajectory leaves the boundary when $\langle n_1(T_b) \rangle = \kappa_b \bar{n}$. The solution to (24) satisfying this is

$$\exp\left(-\delta T_b\right) = \frac{\kappa_0 - \kappa_e}{\kappa_b - \kappa_e}, \tag{25}$$

which requires κ_0. Consider the part of the limit cycle in the interior of the phase space, shown in Supplementary Fig. 1, where R is defined as the distance of the trajectory from the centre of the phase-space,

$$R(t) = \left| \frac{\langle \mathbf{n}(t) \rangle}{\bar{n}} - \frac{1}{3}\left(1, 1, 1\right) \right|. \tag{26}$$

Trajectories in the interior of the phase-space follow exponential spirals so $R(t)$ increases exponentially fast, at a rate δ. Setting $t = 0$ to be the instant at which the trajectory leaves the boundary gives

$$R(T_i) = R(0)\exp(\delta T_i) = R(0)\exp\left(\hat{\theta}\ \omega T_i\right) \tag{27}$$

Using Pythagoras' theorem together with $\kappa_b - \frac{1}{2} = \frac{1}{6}\theta$ gives

$$R(0)^2 = \left(\kappa_b - \frac{1}{2}\right)^2 + \frac{1}{12} = \frac{1}{12}\left(\frac{\theta^2}{3} + 1\right), \tag{28}$$

$$R(T_i)^2 = \left(\frac{1}{2} - \kappa_0\right)^2 + \frac{1}{12}, \tag{29}$$

which from (27) gives

$$\kappa_0 = \frac{1}{2} - \frac{\sqrt{3}}{6}\sqrt{\left(\hat{\theta}^2 + 1\right)\exp\left(2\hat{\theta}\ \omega T_i\right) - 1}. \tag{30}$$

To evaluate T_i note that ω gives the angular frequency and thus ωT_i is the angle swept by trajectory while it moves between the boundaries. Defining the two angles α and β shown in the Supplementary Fig. 1 gives

$$\frac{R(0)}{R(T_i)} = \frac{\cos\beta}{\cos\alpha} = \cos\left(\omega T_i - \frac{2\pi}{3}\right) - \tan\alpha\sin\left(\omega T_i - \frac{2\pi}{3}\right), \tag{31}$$

where we have used $\alpha + \omega T_i + \beta = 2\pi/3$. Recognising that $\tan\alpha = \hat{\theta}$ gives

$$\exp\left(-\hat{\theta}\ \omega T_i\right) = \cos\left(\frac{2\pi}{3} - \omega T_i\right) + \hat{\theta}\ \sin\left(\frac{2\pi}{3} - \omega T_i\right). \tag{32}$$

This equation shows that ωT_i is a function of $\hat{\theta}$, or equivalently θ, with numerical solutions shown in Supplementary Fig. 3(a). From (30), $\kappa_0 = \kappa_0(\theta, \omega T_i) = \kappa_0(\theta)$. Therefore κ_b, κ_e and κ_0 are all functions of the single parameter θ, see Supplementary Fig. 4.

Finally ωT_b is found from (25) to also depend only on θ, the plot of which is given by Supplementary Fig. 3(b). The total time for the trajectory to make one traversal around the limit cycle is given by $3T_i + 3T_b$ and so the frequency of the oscillations in the wave-state is given by

$$f \equiv \frac{1}{3T_i + 3T_b} = r\rho\left[\frac{\sqrt{3}}{6}\frac{1}{\omega T_i + \omega T_b}\right], \tag{33}$$

giving $f(r, \rho, \theta) = r\rho\hat{f}(\theta)$. So the bias ρ for direction of the particle migrations has the effect of scaling time.

Calculating the amplitude

The amplitude A of the limit cycle is defined as the maximum value of the $\langle n_i \rangle / \bar{n}$ over a whole period of the cycle. Supplementary Fig. 2 shows one third of the phase-space, containing the corner where $\langle n_1 \rangle = \bar{n}$. The value of the amplitude is found at a point P_2 where the trajectory is tangential to the lines of constant $\langle n_1 \rangle$. These lines make an angle $\pi/3$ with the two boundaries. As the trajectory is tangent to the boundary when it leaves, the point where $\langle n_1 \rangle = \bar{n}$ will be at an angle $\pi/3$ from the point P_1 where the trajectory leaves the boundary, as shown in Supplementary Fig. 2. Therefore the two triangles OP_1Q_1 and OP_2Q_2 are similar. Hence

$$\frac{\frac{\sqrt{3}}{3} - \frac{\sqrt{3}}{2}(1-A)}{\frac{\sqrt{3}}{6}} = \frac{R\left(\pi/3\omega\right)}{R(0)} = \exp\left(\frac{\pi}{3}\frac{\delta}{\omega}\right), \tag{34}$$

and consequently

$$A = \frac{1}{3}\left[1 + \exp\left(\frac{\pi}{3}\hat{\theta}\right)\right]. \tag{35}$$

There is a value of $\hat{\theta}$ for which the point P_2 meets the boundary where $\langle n_1 \rangle = \kappa_0 \bar{n}$. This occurs when $1 - A = \kappa_0$, or equivalently when $\omega T_i = \pi/3$ which, inserting into (32) gives

$$\exp\left(-\frac{\pi}{3}\hat{\theta}\right) = \frac{1}{2} + \frac{\sqrt{3}}{2}\,\hat{\theta}. \tag{36}$$

The numerical solution to this equation is $\hat{\theta} \approx 0.2821$, or equivalently $\theta \approx 0.4885$. For θ larger than this value, the point P_2 would be found outside of the boundaries of the phase space. It then follows that the amplitude A is found at the point where the trajectory meets the boundary, i.e. $A(\theta) = 1 - \kappa_0(\theta)$.

Calculating θ_c

The critical value of $\theta = \theta_c$ at which the limit cycle can no longer exist corresponds to when $\kappa_0 = \kappa_{ep}$, i.e. the trajectory reaches the boundary at the equilibrium point. Using (29) gives

$$R(T_i)^2 = \left(\frac{1}{2} - \kappa_{ep}\right)^2 + \frac{1}{12} = \left(\frac{1}{2\theta}\right)^2 + \frac{1}{12} = \left(\frac{1}{2\theta}\right)^2\left(1 + \frac{\theta^2}{3}\right). \tag{37}$$

Combining this with (28),

$$\exp\left(-\hat{\theta}\,\omega T_i\right) = \frac{R(0)}{R(T_i)} = \frac{\theta}{\sqrt{3}} = \hat{\theta}. \tag{38}$$

Hence $\hat{\theta}$ can be equated with the right-hand-side of (32) to give

$$\hat{\theta}\left(1-\sin\left(\frac{2\pi}{3}-\omega T_i\right)\right)=\cos\left(\frac{2\pi}{3}-\omega T_i\right), \tag{39}$$

which is true when $\omega T_i = \pi/6$. (38) then gives

$$\frac{\log\hat{\theta}}{\hat{\theta}}=-\frac{\pi}{6}, \tag{40}$$

the solution to which is approximately 0.6950. Multiplying this by $\sqrt{3}$ gives the corresponding value of $\theta = \theta_c \approx 1.2037$.
